# GC and GC/MS Analysis of Essential Oil Composition of the Endemic Soqotraen *Leucas virgata* Balf.f. and Its Antimicrobial and Antioxidant Activities

**DOI:** 10.3390/ijms141123129

**Published:** 2013-11-21

**Authors:** Ramzi A. Mothana, Mansour S. Al-Said, Mohammed A. Al-Yahya, Adnan J. Al-Rehaily, Jamal M. Khaled

**Affiliations:** 1 Department of Pharmacognosy, College of Pharmacy, King Saud University, P.O. Box 2457, Riyadh 11451, Saudi Arabia; E-Mails: msalsaid@ksu.edu.sa (M.S.A.); alyahya@ksu.edu.sa (M.A.A.); ajalreha@ksu.edu.sa (A.J.A.); 2 Department of Pharmacognosy, Faculty of Pharmacy, Sana’a-University, P.O. Box 33039, Sana’a, Yemen; 3 Department of Botany and Microbiology, College of Science, King Saud University, Riyadh 11451, Saudi Arabia; E-Mail: jkhaled@ksu.edu.sa

**Keywords:** *Leucas virgata*, Soqotra, essential oil, antimicrobial, antioxidant

## Abstract

*Leucas virgata* Balf.f. (Lamiaceae) was collected from the Island Soqotra (Yemen) and its essential oil was obtained by hydrodistillation. The chemical composition of the oil was investigated by GC and GC-MS. Moreover, the essential oil was evaluated for its antimicrobial activity against two Gram-positive bacteria, two Gram-negative bacteria, and one yeast species by using broth micro-dilution assay for minimum inhibitory concentrations (MIC) and antioxidant activity by measuring the scavenging activity of the DPPH radical. The investigation led to the identification of 43 constituents, representing 93.9% of the total oil. The essential oil of *L. virgata* was characterized by a high content of oxygenated monoterpenes (50.8%). Camphor (20.5%) exo-fenchol (3.4%), fenchon (5.4%), and borneol (3.1%) were identified as the main components. Oxygenated sesquiterpenes were found as the second major group of compounds (21.0%). β-Eudesmol (6.1%) and caryophyllene oxide (5.1%) were the major compounds among oxygenated sesquiterpenes. The results of the antimicrobial assay showed that the oil exhibited a great antibacterial activity against the tested *S. aureus*, *B. subtilis*, and *E. coli*. No activity was found against *P. aeruginosa* and *C. albicans*. Moreover, the DPPH-radical scavenging assay exhibited only a moderate antioxidant activity (31%) for the oil at the highest concentration tested (1 mg/mL).

## 1. Introduction

Aromatic plants are frequently used in traditional medicine because of their essential oils and volatile constituents. In last few years, there has been an increase in the use of aromatic medicinal plants and their essential oils in scientific research and industrial applications including nutritional, pharmaceutical, and cosmetic uses [1–4]. At present, approximately 3000 essential oils are known, 300 of which are commercially important especially for the pharmaceutical, agronomic, food, cosmetic, and perfume industry [5]. Moreover, various potent biological activities including antimicrobial, antioxidant, anti-inflammatory, and anticancer are attributed to essential oils [6–11]. As essential oils represent a source of antimicrobial, antioxidants and anticancer components, they are currently attracting increasing interest in the scientific community and there is much research being performed on their pharmacological activities, particularly their antimicrobial, antioxidant, anti-inflammatory, and anticancer properties, which are important in the prevention and treatment of diseases of microbial and oxidative stress origin, such as bacterial and viral infections, inflammations, cancers, and cardiovascular diseases, including atherosclerosis and thrombosis [12–19].

The Soqotra Archipelago in Yemen has long been considered the “jewel” of biodiversity in the Arabian Sea. The long geological isolation of the Soqotra archipelago has created a unique and spectacular endemic flora. Surveys have revealed that more than a third of the 800 or so plant species of Soqotra are found nowhere else [20].

The genus *Leucas* (family: Lamiaceae) comprises about eighty species. The highest species diversity has been found in East Africa [21]. The genus *Leucas* is represented in Soqotra Island by ten species including the endemic *Leucas virgata* Balf.f., which occur as widespread and abundant much-branched aromatic shrub, up to 1 m in height [20]. The sprigs and leaves are used as a tea or powder for heartburn and indigestion, as well as to treat a variety of stomach problems [20].

As a part of our phytochemical and biological studies on natural essential oils and their possible antimicrobial and antioxidant activities, the aim of this work was to investigate the chemical composition of the essential oil of one of the endemic traditionally used Lamiaceae species namely, *Leucas virgata*. Moreover, in this study we examined the antimicrobial and the radical scavenging activities of this essential oil.

## 2. Results and Discussion

### 2.1. Chemical Composition of the *L. virgata* Essential Oil

The essential oil of the aerial part of *L. virgata* obtained after hydrodistillation was colorless and possessed an aromatic odor. It gave an average yield of 0.28% on dry weight basis. The chemical composition of the oil is presented in Table 1, in which the compounds are listed in order of their elution on the CP-Sil 5 CB column. The chemical components were grouped in different classes of compounds. A total of 43 constituents, representing 93.9% of the total oil, were identified by gas chromatographic and mass spectrometric data. It is important to mention that there have been no reports on GC-MS analysis of the essential oil of this endemic *Leucas* species. Additionally, this study represents the first report on the antimicrobial and antioxidant activities of the essential oil. *L. virgata* essential oil was characterized by high content of oxygenated monoterpenes (50.8%). Among them, camphor (20.5%), fenchon (5.4%), exo-fenchol (3.4%), and borneol (3.1%) were identified as the main components (Table 1). Oxygenated sesquiterpenes were found as the second major group of compounds (21.0%), of which β-eudesmol (6.1%) and caryophyllene oxide (5.1%) were the major compounds (Table 1). Furthermore, monoterpene hydrocarbons and sesquiterpene hydrocarbons amounted only to 8.1% and 13.8% respectively (Table 1). Based on the data published so far, our results appeared to be somewhat different from previously reported data on the chemical composition of other *Leucas* essential oils.

In previous studies [22–26], the chemical composition of the essential oils of different *Leucas* species e.g., *L. glabrata*, *L. aspera*, *L. milanjiana*, *L. deflexa*, and *L. cephalotes* was investigated. The results obtained by Vagionas *et al.* [22] for the essential oil of *L. glabrata*, revealed that the most abundant chemical category was the oxygenated monoterpenes (64.4%) as well, however, other compounds e.g., menthone (31.8%), pulegone (11.4%), piperitone (10.6%), and piperitenone (6.7%) were the major volatiles. Moreover, the GC-MS analysis of the volatile oil of *L. aspera* identified carvone, carvacrol, menthol, phellandral, and farnesene as major components [23], whereas high content of β-cubebene, α-pinene, *trans*-caryophyllene, and limonene was reported for the essential oil of *L. milanjiana* [24]. These results suggest that phenological stage of the plant as well as geographical environmental factors almost certainly contributed to create a spectacular chemical composition of *L. virgata*.

### 2.2. Antimicrobial Activity

To analyze the antimicrobial activity of the essential oil, the broth microdilution method was employed to determine the minimum inhibitory concentration (MIC) against selected microorganisms. The results of the antimicrobial activity are shown in Table 2. The results exhibited that the essential oil had varying degrees of growth inhibition against the bacterial strains (Table 2). In general, the tested Gram-positive bacterial strains showed more susceptibility to the investigated essential oil than the Gram-negative bacterial strains. The essential oil of *L. virgata* showed a strong antimicrobial activity against *B. subtilis*, *S. aureus*, and *E. coli* with MIC values of 0.28, 0.56, and 4.5 mg/mL, respectively (Table 2). However, no obvious inhibitory activity was observed against *P. aeruginosa* and *C. albicans*. Obviously, our result is in agreement with data reported on the antimicrobial effect of essential oils as well as methanolic extracts from other *Leucas* species [22,23,27,28]. Vagionas *et al.* [22] reported that the tested essential oil of *L. glabrata* has a strong antimicrobial activity against two Gram-positive and four Gram-negative bacteria, three pathogenic fungi, and two oral pathogens (MIC values 0.45–1.14 mg/mL). It was reported that the antimicrobial activity could be attributed to the high content of the oil in compounds with known antimicrobial activity, such as menthone [29], pulegone, and piperitone [30]. Moreover, Satyal *et al.* [31] recently reported that the essential oil of *L. aspera* exhibited no activity against *E. coli*, *P. aeruginosa*, and *C. albicans* (MIC > 1250 μg/mL) but good activity against *S. aureus* (625 μg/mL) and *B. cereus* (313 μg/mL). A similar study was conducted by Gerige *et al.* [23] who reported similar results for *L. aspera*. Schmidt *et al.* [32] and Satyal *et al.* [31] attributed this effect to the high percentage of sesquiterpenes such as (*E*)-caryophyllene and α-humulene, which have shown antibacterial activity against *S. aureus* and *B. cereus*. In addition to that, oxygenated monoterpenes, such as camphor, borneol, linalool, and α-terpineol, were reported to be responsible for the antimicrobial activity of several essential oils [33–35]. Consequently, the observed strong antibacterial effect of *L. virgata* might be attributed to the high percentage of oxygenated monoterpenes, such as camphor, α-fenchol, fenchon, borneol, and α-terpineol. Possible synergistic effect of some compounds in the oils e.g., oxygenated sesquiterpenes (caryophyllene oxide, humulene oxide II, β-eudesmol, γ-eudesmol, and β-bisabolol) should also be taken in consideration.

### 2.3. Antioxidant Activity

The potential antioxidant activity of the essential oil was determined on the basis of scavenging activity of the free radical DPPH. The investigated essential oil of *L. virgata* demonstrated only moderate antioxidant abilities to reduce DPPH (Table 2) at the highest concentration tested (31% at 1 mg/mL). This observed effect could be associated with low content of volatile phenolic components, such as thymol and carvacrol in the oil [36]. This result seems to be in agreement with results reported by Vagionas *et al.* [22] who demonstrated that the tested essential oil of *L. glabrata* has a weak antioxidant activity. It is interesting to point out that we previously investigated the antioxidant activity of the methanolic extract of *L. virgata* [37]. The methanolic extract showed high radical scavenging activity (80%) at 1 mg/mL. In agreement with that result, remarkable antioxidant activity was reported for the methanolic and ethanolic extracts of other *Leucas* species, such as *L. mollissima* and *L. aspera* [38,39]. Recently, similar results were published for extracts of *L. aspera*, *L. martinicensis*, and *L. lanata* [40–42]. It was shown that these extracts had strong antiradical scavenging activity [38–42]. It was found that the interesting antioxidant effect is attributed to the presence of flavonoids and other phenolic compounds [42,43]. Thus, the notable antioxidant activity of the methanolic extract of *L. virgata* should be attributed to the phenolic and flavonoidal content [37].

## 3. Experimental Section

### 3.1. Plant Material

The aerial part (including flowers, leaves, and stems) of the plant was collected from the island Soqotra in February 2007, and identified at the Pharmacognosy Department, Faculty of Pharmacy, Sana’a University. Voucher specimens were deposited at the Pharmacognosy Department, Faculty of Pharmacy, Sana’a University.

### 3.2. Isolation of the Essential Oil

The air-dried and ground aerial part of *L. virgata* was submitted for hydrodistillation (3 h) in a Clevenger-type apparatus according to the European Pharmacopoeia. As a collector solvent, 1.5 mL of *n*-heptane was used. The obtained essential oil was dried over anhydrous sodium sulphate and after filtration and evaporation under nitrogen-flow, stored in sealed vials at +4 °C until tested and analyzed.

### 3.3. Gas Chromatography Analysis

The volatile oil was analyzed using a Hewlett Packard 5890 series II GC equipped with a Flame Ionization Detector (FID). The analysis was carried out on a fused silica capillary CP-Sil 5 CB column (Agilent Technologies, Santa Clara, CA, USA) (30 m × 0.25 mm i.d., film thickness 0.25 μm). Nitrogen was used as a carrier gas at a flow rate of 0.46 mL/min. Injector and detector temperature were set at 200 °C and 280 °C, respectively. Oven temperature was kept at 45 °C then gradually raised to 280 °C at 3 °C/min and finally held isothermally for 22 min. One microliter of the diluted samples (1/100 in heptane, *v*/*v*) were injected manually (split mode, split ratio 1:16). Calculation of peak area percentage was performed on basis of the FID signal using the GC HP-Chemstation software (Agilent Technologies, Santa Clara, CA, USA).

### 3.4. Gas Chromatography-Mass Spectrometry

The GC–MS analysis of the oil was performed on a Hewlett-Packard 5890 gas chromatograph, coupled to VG Analytical 70-250S mass spectrometer. The GC was equipped with a fused silica capillary CP-Sil 5 CB column (25 m × 0.25 mm i.d., film thickness 0.40 μm, from Chromback, Varian). Helium was used as carrier gas at flow rate of 1 mL/min. The oven program started with an initial temperature of 80 °C held for 2 min and then the oven temperature was heated at 10 °C/min to 270 °C and finally held isothermally for 20 min. For GC-MS detection, an election ionization system, with ionization energy of 70 eV was used. A scan rate of 0.6 s (cycle time: 0.2 s) was applied, covering a mass range from 35 to 600 amu.

### 3.5. Identification of Components

The identification of the compounds was based on the comparison of retention indices and mass spectra of most of the compounds with data generated under identical experimental conditions by applying a two-dimensional search algorithm, considering the retention index, as well as mass spectral similarity [44,45], or with those of authentic compounds available in our laboratories. Moreover, special software, namely MassLib software (V9.3-106; 1996–2008, Max-Planck-Institute for Kohlenforschung, Muelheim, Germany) was used for processing and interpretation of mass spectra with several commercially available libraries included: Wiley Registry of Mass Spectral Data (4th Ed.), NIST/EPA/NIH Mass Spectral Library (2005), Library MPI Mühlheim (2006), Geochemicals (1st Ed.), MRC collection (1st Ed.), and CC (4th Ed.) [45]. The retention indices (RI) were in relation to a homologous series of *n*-alkanes (C_6_–C_28_) on the CP-Sil 5 CB column under the same chromatographic conditions. Components relative concentrations were obtained by peak area normalization. No response factors were calculated.

### 3.6. Determination of Antimicrobial Activity

#### 3.6.1. Test Organisms

The following microorganisms were used as test organisms in the screening: *Staphylococcus aureus* (BNI 18), *Bacillus subtilis* (BNI 28), *Escherichia coli* (BNI 2), *Pseudomonas aeruginosa* (BNI 20), and *Candida albicans* (BNI 33). The microbial strains were obtained from the Bernhard-Nocht-Institute (BNI) for Tropical Medicine, Hamburg, Germany.

#### 3.6.2. Broth Micro-Dilution Assay for Minimum Inhibitory Concentrations (MIC)

The antimicrobial activity of the essential oil was determined by the broth micro-dilution method described by Mann and Markham [46] with modifications against the above-mentioned microbial strains. With sterile round-bottom 96-well plates, duplicate two-fold serial dilutions of extract (100 μL/well) were prepared in the appropriate broth containing 5% (*v*/*v*) DMSO. Two-fold dilutions of amoxicillin, gentamicin, or nystatin were used as a positive control. A bacterial cell suspension (prepared in the appropriate broth) of 100 μL, corresponding to 1 × 10^6^ CFU/mL, was added in all wells except those in columns 10, 11, and 12, which served as saline, essential oil, and media sterility controls, respectively. Controls for bacterial growth without essential oil were also included on each plate. The final concentration of bacteria in the assay was 5 × 10^5^ CFU/mL. Plates were then incubated at 37 °C for 18 h overnight. After incubation, the MIC of each essential oil was determined as the lowest concentration at which no growth was observed in the duplicate wells. Twenty microliters of a *p*-iodonitro-tetrazolium violet solution (0.04%, *w*/*v*) (Sigma, St. Louis, MO, USA) was then added to each well. The plates were incubated for a further 30 min, and estimated visually for any change in color from yellow to pink, indicating reduction of the dye due to bacterial growth. The highest dilution (lowest concentration) that remained yellow corresponded to the MIC. Experiments were performed in duplicate.

### 3.7. Determination of Antioxidant Activity (Scavenging Activity of DPPH Radical)

The DPPH free radical scavenging assay was carried out for the evaluation of the antioxidant activity. This assay measures the free radical scavenging capacity of the investigated essential oil. DPPH is a molecule containing a stable free radical. In the presence of an antioxidant which can donate an electron to DPPH, the purple color, typical for free DPPH radical decays, and the change in absorbency at λ = 517 nm is followed spectrophotometrically. This test provides information on the ability of a compound to donate a hydrogen atom, on the number of electrons a given molecule can donate, and on the mechanism of antioxidant action. The method was carried out as described previously [47]. The essential oil was dissolved in methanol, and various concentrations (10, 50, 100, 500, and 1000 μg/mL) were used. The assay mixture contained in a total volume of 1 mL, 500 μL of the oil, 125 μL prepared DPPH (1 mM in methanol), and 375 μL solvent (methanol). After 30 min incubation at 25 °C, the decrease in absorbance was measured at λ = 517 nm. The radical scavenging activity was calculated from the equation:

(1) % of radical scavenging activity=Abscontrol-Abssample/Abscontrol×100

## 4. Conclusions

In conclusion our study is the first report on the chemical composition and *in vitro* antimicrobial and antioxidant activities of the essential oil of the Soqotraen *Leucas virgata*. The GC and GC/MS analysis of the essential oil revealed that the chemical composition was characterized by high content of oxygenated monoterpenes (50.8%). Camphor, exo-fenchol, fenchon, and borneol were identified as the main components. It was shown that the chemical compositions differed from that of other well-investigated *Leucas* species. Moreover, the results clearly showed that the essential oil of *L. virgata* possesses potent antimicrobial but moderate antioxidant activity. Our results further support the idea that *L virgata* can be promising source of potential antimicrobial agents.

## Figures and Tables

**Table 1:** Chemical composition of the essential oil *Leucas virgata*.

No.	Compounds	RI	% Occurrence	Identification
1	α-Pinene	932	1.7	1,2
2	Camphene	948	1.8	1,2
3	Sabinene	967	0.1	1,2,3
4	β-Pinene	975	2.3	1,2,3
5	2-Pentylfuran	982	0.3	1,2,3
6	α-Phellandrene	998	0.3	1,2,3
7	*p*-Cymene	1014	0.5	1,2
8	Limonene	1024	1.0	1,2,3
9	γ-Terpinene	1051	0.2	1,2,3
10	*t*-Linalool oxide	1059	1.2	1,2
11	Fenchone	1070	5.4	1,2
12	Linalool	1085	2.7	1,2,3
13	α-Fenchol	1102	2.0	1,2,3
14	Exo-Fenchol	1111	3.4	1,2
15	Camphor	1125	20.5	1,2
16	Pinocarvone	1142	2.1	1,2
17	Borneol	1152	3.1	1,2,3
18	Terpinen-4-ol	1165	2.0	1,2,3
19	α-Terpineol	1176	3.0	1,2
20	Myrtenol	1182	2.9	1,2,3
21	*trans*-Carveol	1201	0.9	1,2
22	*cis*-Carveol	1213	0.2	1,2
23	Carvone	1219	0.6	1,2
24	*t*-Geraniol	1236	0.1	1,2
25	Bornyl acetate	1272	0.2	1,2
26	Carvacrol	1281	0.4	1,2,3
27	Eugenol	1333	0.1	1,2
28	(*E*)-β-Caryophyllene	1423	0.7	1,2,3
29	*trans*-Bergamotene	1434	0.4	1,2
30	α-Humulene	1456	1.6	1,2
31	β-Ionene	1463	0.6	1,2
32	γ-Muurolene	1475	2.9	1,2
33	β-Selinene	1488	2.4	1,2
34	β-Curcumene	1502	1.8	1,2
35	α-Alaskene	1513	1.7	1,2
36	α-Calacorene	1535	1.7	1,2
37	Caryophyllene oxide	1580	5.1	1,2
38	Humulene epoxide II	1604	3.1	1,2
39	γ-Eudesmol	1623	3.4	1,2
40	β-Eudesmol	1646	6.1	1,2
41	Isoaromadendrene epoxide	1663	0.4	1,2
42	β-Bisabolol	1674	2.9	1,2
43	*n*-Tetradecanoic acid	1745	0.2	1,2
	Monoterpene hydrocarbons		8.1	–
	Oxygenated monoterpenes		50.8	–
	Sesquiterpene hydrocarbons		13.8	–
	Oxygenated sesquiterpenes		21.0	–
	Aliphatic acids		0.2	–
	Total		93.9	–

Notes: RI, retention indices relative to C8–C30 n-alkanes on the CP-Sil 5 CB column, tr: traces, 1: retention index, 2: mass spectrum, 3: co-injection with authentic compound.

**Table 2:** Antimicrobial activity (minimum inhibitory concentration (MIC)-values) and free radical scavenging activity of the investigated essential oil of *L. virgata*.

Plant species	Radical scavenging activity in %					MIC a				
	
	10 (μg/mL)	50 (μg/mL)	100 (μg/mL)	500 (μg/mL)	1000 (μg/mL)	*S. aureus*	*B. subtilis*	*E. coli*	*P. aeruginosa*	*C. albicans*
*L.virigata*	5.9	6.1	10.4	18.4	31.0	0.56	0.28	4.48	–	–
Amoxicillin	–	–	–	–	–	3.5	3.5	nt	nt	nt
Gentamicin	–	–	–	–	–	nt	nt	3.5	7.0	nt
Nystatin	–	–	–	–	–	nt	nt	nt	nt	3.5
Ascorbic acid	43.5	84.3	92.6	96.5	95.2	–	–	–	–	–

Notes:

a minimum inhibitory concentration values are given as mg/mL for essential oils and μg/mL for standard antibiotics,

nt: not tested.
